# Adults who stutter lack the specialised pre-speech facilitation found in non-stutterers

**DOI:** 10.1371/journal.pone.0202634

**Published:** 2018-10-10

**Authors:** Alexander Whillier, Sina Hommel, Nicole E. Neef, Alexander Wolff von Gudenberg, Walter Paulus, Martin Sommer

**Affiliations:** 1 Department of Clinical Neurophysiology, University Medical Centre Göttingen, Göttingen, Germany; 2 Department of Neuropsychology, Max Planck Institute for Human Cognitive and Brain Science, Leipzig, Germany; 3 Institut der Kasseler Stottertherapie, Bad Emstal, Germany; University of Zurich, SWITZERLAND

## Abstract

**Objectives:**

Persistent developmental stuttering is a speech fluency disorder defined by its symptoms, where the underlying neurophysiological causes remain uncertain. This study examined the underlying neurophysiological mechanisms of the speech planning process, using facilitation in the motor cortex during speech preparation as an analogue.

**Methods:**

transcranial magnetic stimulation (TMS) pulses induced motor evoked potentials (MEPs), which were recorded from the tongue. Eighteen adults who stutter (AWS) and 17 adults who do not stutter (ANS) completed three experiments, which involved reading a German prefix+verb utterance from a screen. Each experiment involved 120 trials with three distinct levels of speech production: *immediate* speech, *delayed speech without pacing* and *delayed speech with predefined pacing*. TMS was applied shortly before speech onset. Trial MEPs were normalised to average non-speech MEPs. MEP amplitude, MEP facilitation ratio (amplitude: pre-speech offset) and group difference were the outcomes of interest analysed by multiple regression, as well as speech reaction time analysed by correlation.

**Results:**

MEP values were 11·1%-23·4% lower in AWS than ANS (by standardised Beta), across all three experiments. MEP facilitation ratio slopes were also 4·9%-18·3% flatter in AWS than ANS across all three experiments. Reaction times for AWS were only significantly slower than for ANS in *immediate* speech and *predefined pacing* experiments. No stuttering was detected during the trials. The group difference in *immediate* speech was 100% and 101% greater than the other two experiments respectively.

**Discussion:**

While performance of both ANS and AWS worsens under disturbed speech conditions, greater disturbance conditions affected controls worse than AWS. Future research and therapy in stuttering should focus on non-disturbed speech.

## 1.0.0 –Introduction

Fluent and effortless speech production is one of the most elaborate skills that humans have evolved; it is one of the most common human functions, yet it is often only remarkable when it is disturbed. Stuttering, one of the most common speech disturbances, has two main categories–Childhood-Onset Fluency Disorder [1] and Acquired/Neurogenic stuttering. As the names suggest, the former begins in childhood but persists through to adulthood, while the latter is the result of trauma (e.g. stroke). This study focused on the former, which we refer to hereafter simply as stuttering.

As a speech fluency disorder, stuttering is characterised by intermittent involuntary interruptions of speech motor control. The interruptions can occur in the form of repetitions of speech sounds, prolongations, and tense pauses, among other symptoms (e.g. [1]). This has the potential to affect psychological wellbeing and development, as well as having impacts both academically and culturally. Additionally, stuttering can be influenced by various factors–e.g. increased when stressed/anxious, reduced when planned/rehearsed–and it can even disappear completely during external pacing–e.g. singing [2]. According to the diagnostic criteria, it is estimated that around 5% of children stutter during language development–typically before age 6 [3]. However, ongoing neurological development in these children results in spontaneous recovery in around 80% of such cases. As a result, stuttering in the general population is estimated at around 1% of adults, mostly males, with equivalent levels reported in many countries [4].

To date, stuttering is defined by its symptoms, as the cause remains under debate. While there have been many imaging studies examining the structural neurological differences between Adults Who Stutter (AWS) and Adults who do Not Stutter (ANS), the findings are varied across the cortex. Among the most robust findings are that of a reduction in the white matter integrity of left hemispheric speech motor regions [5–8], recently confirmed by an ALE meta-analysis [9], and a deficit in the left inferior frontal-premotor functional-connectivity [10–12]. In recovered AWS, the findings are reduced but still present as compared with ANS, both in children [13] and in adults [7]. This suggests that, beyond purely structural differences, stuttering as a disorder is also governed by differences in both functional activity and brain plasticity.

Earlier work by Brown and colleagues [14] identified imbalanced activation in the speech-related auditory and motor cortices, which encouraged further examination of the local excitability of the speech-motor areas of the brain. Excitability regulation of cortical neurons forms the basis of motor action sequences, underlying the planning and execution behind smooth coordinated motion [15]. As such, according to this theoretical explanation, a systematic mismatch exists in the primary motor cortex between the facilitation impulse signals and the inhibition of neural populations [16], and it posits that this mismatch causes spontaneous undesired movement, such as stutter symptoms. Recent evidence supports the idea of reduced inhibitory motor control in AWS in non-speech tasks [17] and in speech motor tasks [12]. Busan and colleagues [18] postulated that a lack of left hemisphere dominance in motor cortical excitability for speech activation was the likely explanation behind their findings of reduced neural activations in AWS.

Immediately before planned movement onset, neural activity spikes; this can be detected in the pre-movement period by Motor Evoked Potentials (MEPs)–potentials recorded by EMG, in the low mV range, following an external stimulation. Transcranial Magnetic Stimulation (TMS) is a non-invasive stimulation technique often employed in the examination of MEPs in stuttering [18–20]. TMS works by generating a focussed electromagnetic pulse, capable of stimulating a small section of cerebral cortex–approximately 1cm^3^ –in a single burst of a few microseconds [21, 22]. In using this technique, most studies examine peripheral MEPs–typically at the hand or wrist–and infer that their findings apply similarly to the speech motor cortex when testing AWS. More recently, however, Neef and colleagues [23, 24] took this examination further by examining the orofacial muscles–the muscles associated with speech–in an examination of speech motor cortex excitability.

In their recent study, Neef and colleagues used TMS to elicit MEPs in the tongue during speech production [24]. They first demonstrated that, although there is equivalent bilateral innervation of the orofacial muscles on both sides, there is significantly greater left hemispheric excitability compared with the right hemisphere in fluent speakers during speech. They [24] then demonstrated that this left hemispheric facilitation was absent in stutterers. Further, they found an inverse correlation between stuttering severity and the facilitation level, implying that this is a possible pathophysiological candidate behind stuttering. Overall, this [24] gave a greater understanding of the role of cortical excitability in stuttering. Nonetheless, the chosen utterance was short (a single verb plus particle), with externally regulated timing and pacing and an equivalent level of planning in every trial. However, as previously mentioned, this can alter stuttering severity [2] and may not reflect normal speech conditions.

Indeed, increased cognitive load has long been identified as a factor in stuttering frequency (e.g. [25]). It has also been suggested that concurrent activity during speech can adversely affect AWS with low working memory capacity (see [26]). While the concurrent coordination of the articulators during spontaneous speech may involve an increased cognitive load, the previous setup [24] of externally regulated speech could be seen as increasing the task complexity. We therefore expanded upon the previous design in order to address these two alternate interpretations of cognitive load in stuttering.

To represent unregulated (spontaneous) and mildly disturbed speech within the confines of the previous experimental setup, we designed two new patterns–*immediate speech* (exp. 1) and *delayed speech without pacing* (exp. 2)–as well as recreating the previous experiment [24] of *delayed speech with pacing* (in exp. 3). In the first two experiments, we planned the variations in the cortical and myogenic states to illustrate the different aspects of disruption that occur during speech preparation. For simplicity, this study considered the differences between these three experimental conditions as representative of differences in working memory complexity.

We first hypothesised that:

1. MEP facilitation would increase before speech–represented by a positive correlation between peak-to-peak MEP facilitation and MEP Time before Speech Onset (MEP-TSO), in all experiments;
2. AWS would exhibit a reduction in overall facilitation in each experiment, compared with ANS;
3. AWS would exhibit reduced facilitation over time–represented by an interaction effect between group and MEP-TSO; and that
4. the between group differences would be greatest when cognitive load intensity is high.

Neef and colleagues [24] also raised the issue that stuttering events would not be accurately detectable with the mouthpiece inserted. Rather, they used reaction time (RT) to represent fluent speech production in the context of this test, as most stuttering occurs at word onset [4]. Neef and colleagues [24] found no difference in RTs, which they interpreted to indicate that their participants did not stutter during the experiment. It has been shown that the insertion of a mouthpiece can improve fluency in AWS [27], which may explain these findings. However, it has also long been known that AWS have slower reaction time (RT) on speech tasks but not on other RT tasks (e.g. [28]). Additionally, we anticipated a level of distraction caused by the TMS pulse as TSO diminishes. We suspected that all of these factors would influence RT. Therefore, we also included reaction time as a variable of interest.

Additionally, as with many developmental disorders, it has been suggested that stuttering exists on a continuum [29], rather than as a categorical diagnosis. Therefore, we ran post-hoc analyses to examine whether speech fluency would better represent the between-groups hypotheses above.

Thus we had two secondary hypotheses:

5 that AWS would respond slower on average than ANS across all three experiments;
6 that ‘percentage of stuttered syllables’ (%SS), irrespective of group, would better correlate with MEP facilitation than the current method of between-groups analyses.

## 2.0.0 –Materials and methods

### 2.1.0 –Participants

For the present study, we recruited 18 stuttering speakers (two females) as well as 17 fluent speaking controls (eight females). All participants were native monolingual or bilingual speakers of German. Stuttering participants were recruited from the local stuttering support group in Göttingen, from the nearby Kassel Stuttering Therapy centre and by advertising on bulletin boards in the university in Göttingen. Fluent speakers were also recruited by advertisement around the university.

Demographic details, including age, sex, handedness, education and relevant family history, as well as stuttering severity and motor threshold, were gathered for all participants at the onset of the testing session. The AWS had a mean age of 26.17 years (SD = 8.36) and the controls had a mean of 24.00 years (SD = 3.31); both groups were predominantly right handed and the groups did not differ by education ranks (see Table 1 for demographic information and the supplemental file S1 Table for data for each individual). There was a significant difference between the sexes in each group (χ2 = 6·429, p = ·011) due to complications during recruitment, however this was not deemed unlikely to affect the experiment.

**Table 1:** Demographic data.

Measures	Stuttering	Controls	Significance
Participants, n	18 (16M, 2F)	17 (9M, 8F)	p = ·011 (sig.)
Age in years, mean	27·11 (SD = 9·04)	24 (SD = 3·32)	p = ·185 (n.s.)
Handedness, mean	78·07 (SD = 51·76)	85·96 (SD = 16·99)	p = ·547 (n.s.)
Education, mean rank	2·67 (MR = 14·81)	3·65 (MR = 21·38)	p = ·057 (n.s.)
Motor threshold left hemisphere, mean	45·56 (SD = 7·39)	44·80 (SD = 4·23)	p = ·741 (n.s.)
Percentage of syllables stuttered, mean	10·80 (SD = 11·28)	0·62 (SD = 0·00)	p<·001 (sig.)
SSI-3 Mean Score	23·33 (SD = 11·60)	4·29 (SD = 2·49)	p<·001 (sig.)
Severity Assessment	Moderate	None	-

All group differences were calculated by t-test except for *Education*, which was calculated by Mann-Whitney U test; *U* = 95.5, *z* = -1.93, *p* = .057. Education was assessed on an ordinal scale (1 –high school to year 10; 2 –high school to year 13; 3 –<2 years university; 4–2+ years university; 5–4+ years university and graduated; 6 –completed doctorate); all other variables were scalar. Handedness was assessed with the Edinburgh Handedness Inventory, translated (Schwarz et al., 1995)–handedness is assessed between -100 (completely left handed) and 100 (completely right handed). SD = standard deviation. MR = mean rank.

Stuttering severity of both fluent and stuttering participants was assessed by a speech language pathologist, using the Stuttering Severity Index 3 or SSI-3 ([30]; German adaption [31]). We collected two video recordings of speech from each participant–reading aloud from a sample text of 500 syllables in length, and spontaneous speech elicited by a standard interview. The SSI-3 uses the frequency and duration of stuttered syllables, as well as physical concomitants of stuttering to score each participant. Despite detecting the small instances of speech pauses and dysfluencies associated with normal speech in all participants, all of the fluent speakers were classified as non-stuttering (SSI-3 overall score <10). Among the AWS participants, two were also classified as non-stuttering; nonetheless, as these participants had past diagnoses of stuttering by qualified speech language pathologists and due to the fact that stuttering severity can vary considerably based on situation and emotional state, we included these two participants in the AWS group to represent low severity stuttering (see supplemental file S1 Table for an individual breakdown).

Seven AWS reported a family history of stuttering. None of the fluent speakers reported having a family history of speech or language disorders. Before commencement of TMS stimulation, all participants were screened for inclusion based on the criteria for standard TMS safety screening [32]–due to the magnetic nature of the device, the questionnaire includes past medical history of the head, surgeries, and work history relevant to metal objects. All participants were deemed fit and able to continue. Besides stuttering in the test group, none of the participants reported a history of neurological disease nor did they show any signs of neurological deficits in a routine neurological examination. Additionally, no participants reported any other medical condition or drug use that would impact the experiment. All participants gave their written informed consent to participate in the study. The protocol used in this study was approved by the Institutional Review Board of the University Medical Centre Göttingen.

### 2.2.0 –Electromyography

**Fig 1:**
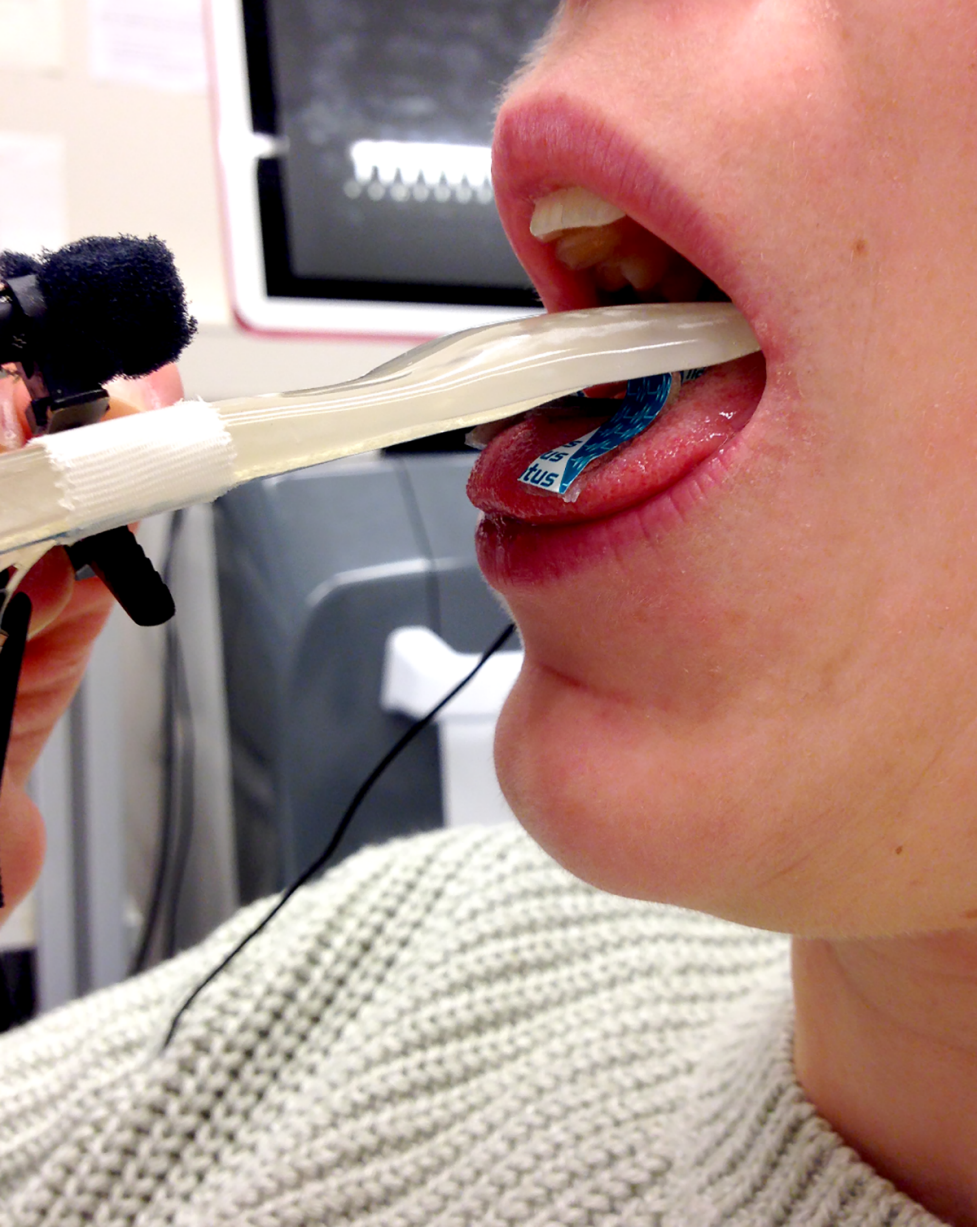
Tongue setup. The electrodes are secured to the underside of the mouthpiece and rest on the tongue. The microphone is also attached to the mouthpiece, visible on the left.

For the EMG recordings, participants sat comfortably relaxed in a reclining chair. We made surface recordings of the lingual muscle bilaterally and simultaneously on both sides of the tongue with two pairs of disposable, pre-gelled, silver/silver chloride (Ag/AgCl) ring electrodes (5mm x 100mm, Viasys Neurocare). The electrodes were mounted in a custom made spoon-shaped silicon mouthpiece produced with dental-laboratory technology. Participants placed the mouthpiece in the mouth, resting on the upper surface of the tongue. They were asked to close their lips and teeth softly around the mouthpiece without additional pressure and, if necessary, to hold the end of the mouthpiece with the left hand and their elbow resting; this ensured that their active hand was ipsilateral to the TMS stimulation site. During the recordings, the participants were asked to raise the tongue against the electrodes (see Fig 1) and their inferior teeth–this procedure was an update of a previous technique [23, 24, 33].

Each recording consisted of two surface EMG signals and one audio signal. The EMG signals were acquired using a Digitimer D360 at a sampling frequency of 5 kHz and amplified (x1000), then filtered (Butterworth bandpass filter 20 Hz to 2kHz) using a 1401 laboratory interface (Cambridge Electronic Design mikro 1401 mk II, UK). Recordings were captured by Signal Software (Cambridge Electronic Design, version 2.16). For the audio signal, we attached a wireless microphone (AKG PT 40) to the mouthpiece and fed the acquired audio signal into a third channel of the CED Mikro 1401, in order to convert the analog signal into a digital one, and in order to ensure that the EMG and audio recordings were temporally matched.

### 2.3.0 –Transcranial magnetic stimulation (TMS)

**Fig 2:**
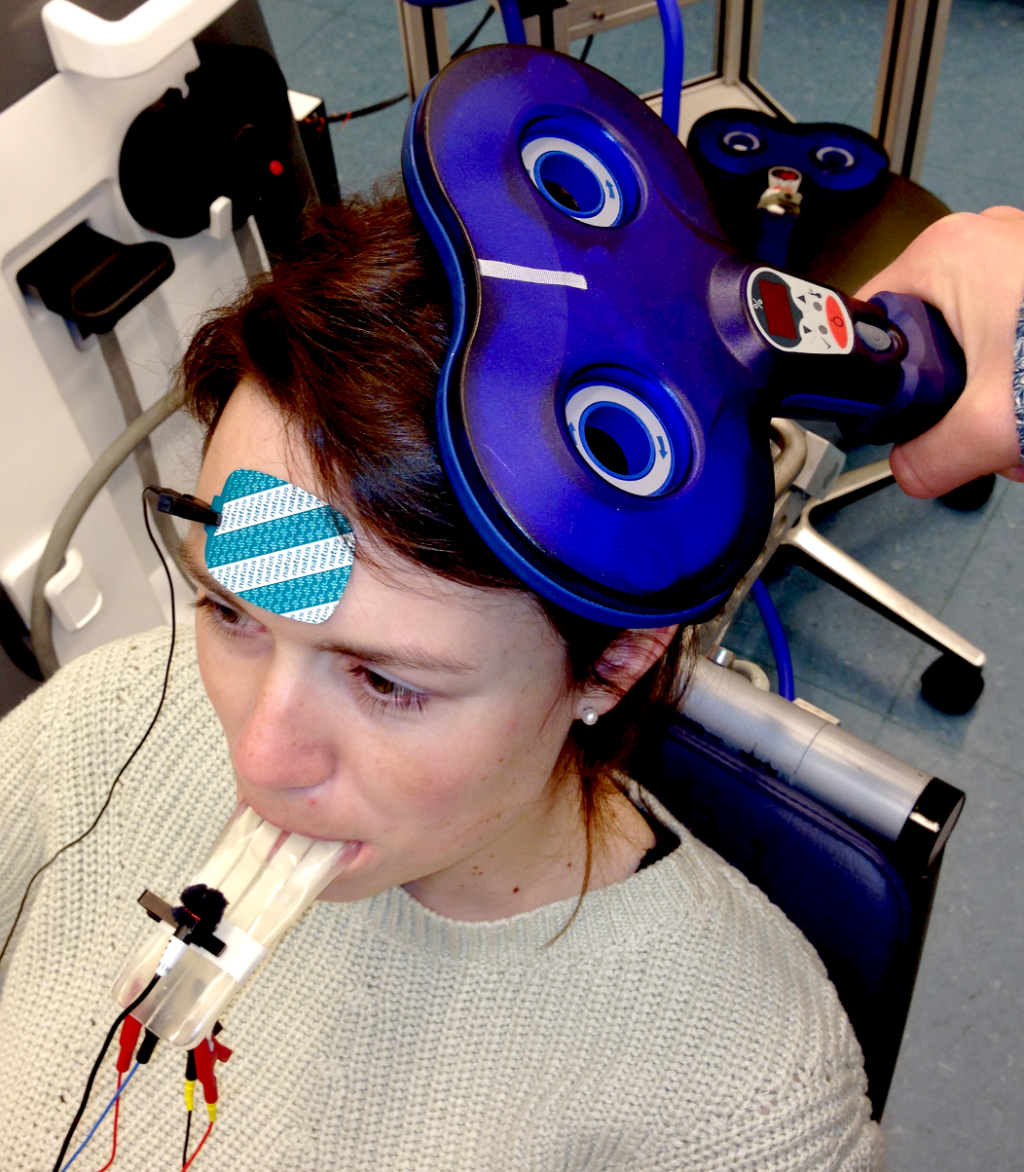
TMS setup. The mouthpiece with electrodes rests on the tongue, with ground electrode on the forehead. The microphone is clipped to the mouthpiece. TMS stimulator is held perpendicular to the scalp over the M1, oriented at 45° to the sagittal and frontal planes. The individual depicted has given written informed consent to be depicted here.

We used a Magstim 200^2^ magnetic stimulator with a monophasic current waveform (Magstim company) to apply single-pulse TMS of the primary motor cortex with a standard figure-of-eight coil with mean loop diameter of 7 cm. The coil was positioned tangentially to the skull, laterally at an angle of 45° to the sagittal plane (see Fig 2); the handle pointed backwards to generate posterior-anterior direction current flow in the brain [34, 35]. The optimum scalp position was marked when the stimulation elicited the largest motor response. To find the optimal position of the coil, we explored the scalp surface systematically. We defined the ‘hot spot’ as the position that consistently induced maximal MEPs in the contralateral tongue site, while at lowest stimulus strength; this was marked on the scalp with a pen to ensure accurate coil placement throughout the experiment [23, 36]. The interstimulus interval between single TMS pulses was 6s (+/-10%, ~0.2Hz).

A maximum of 30 pulses was applied before replacing the self-adhesive electrodes–due to salivation, the electrodes had to be regularly optimised. The participants rested during the cleaning process, which took approximately 5 minutes. A maximum of 100 pulses was applied to determine the hotspot and the motor threshold. In each participant, the hotspot of the motor tongue area was found approximately 2–3 cm anterior and 1–2 cm lateral to the hand representation, consistent with the literature [37].

To determine the motor threshold, we applied single TMS pulses and found the minimal stimulus intensity (to the nearest 1% of maximum stimulator output) required to produce MEPs *of greater than 100μV* in at least three of six consecutive stimuli. For this experiment, the trials were set to 120% of the motor threshold.

TMS allows a high level of temporal accuracy for a given pulse, but stimulation can only be reliably delivered once every few seconds. As the reaction time of each participant for each trial was unpredictable, one pulse was delivered per trial. Each trial was quasirandomly assigned to one of many pulse timings, balanced within each participant and experiment (see Experiment Setup). This ensured that the resultant data adequately covered the 500ms prior to the moment of speech onset. Additionally, some pulse timings were not set to overlap with speech onset, for use in comparing baseline values. Finally, some trials were assigned as control trials, which had no pulse.

### 2.4.0 –Verbal stimuli and speech task

The verbal stimuli were randomly drawn from a list of 49 German verbs–each verb selected had a consonant cluster onset, and each verb was preceded by the German initial particle “auf”, for example “auf-stehen” (to stand up). The verbs were adapted from the previous paper [24] and were used for all three experiments. In all three experiments, the participant read out the full form of prefix+verb. Each participant completed all three experiments. The experiments were performed in order, on the same day for each participant.

In order to investigate the group differences between unprepared speech and prepared speech, we designed each experiment to maintain equivalent cortical state (neurological excitability) and myogenic state (tongue activity) in both groups. The use of the prefix “auf” was planned in the previous experiment [24]; we maintained this design for comparability. Experiment 3 reproduced the previous design, while experiments 1 and 2 varied the cognitive load from this design in order to represent alternative versions of speech onset disturbance.

Within each experiment, the order of the verbs was randomised between participants; additionally, the order of pulse timings was randomly assigned for each participant. This ensured that participants could not anticipate the timing of each pulse.

### 2.5.0 –Experiment setup

#### 2.5.1 –First experiment–immediate speech

**Fig 3:**
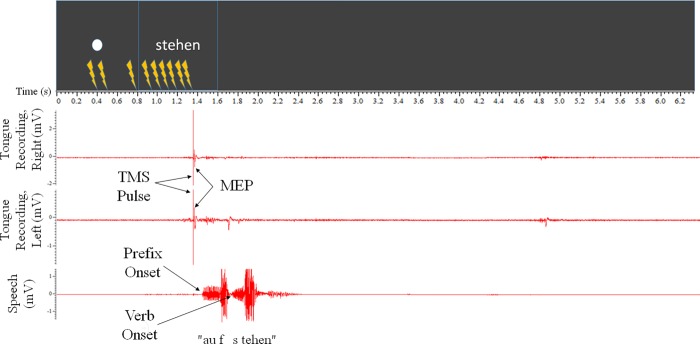
Example trial from Experiment 1. From the participants’ perspective, each trial began with the “alertness” signal of the white circle for 800ms. The verb was then presented for 800ms; this was then followed by 4700ms of blank screen, for a total of 6300ms. The participant spoke the prefix+verb utterance when the verb was presented. One TMS pulse would fire during each trial. Within this design, there were nine possible TMS pulse timings– 400ms, 500ms, 800ms, 960ms, 1040ms, 1120ms, 1200ms, 1280ms and 1360ms (each represented by the stimulation symbol)–as well as a null pulse condition.

In the first experiment, the participant had to immediately speak the prefix and verb, as soon as the verb appeared (see Fig 3); this design maximised the cognitive load of motor planning. The participants were instructed that a large white fullstop character would signal “readiness” directly prior to the target verb and that they should respond with the required “auf+verb” utterance for each verb that appeared.

Each trial was 6300ms long; however, the presentation on the screen was continuous for the two blocks of 60 trials, with only a short pause between the two blocks. See Fig 3 for an example trial.

#### 2.5.2 –Second experiment–delayed speech without pacing

**Fig 4:**
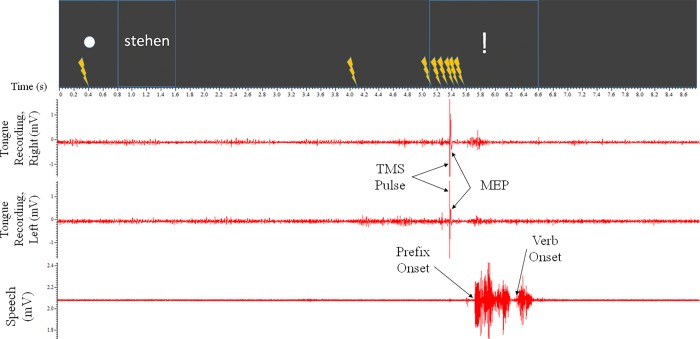
Example trial from Experiment 2. From the participant’s perspective, each trial began with the “alertness” signal of the white circle for 800ms. This was followed by a verb for 800ms, then a blank interval of 3500ms. The “go” signal of an exclamation mark was then presented for 1500ms. This was then followed by a blank screen for 2200ms, for a total trial length of 8800ms. Participants spoke the prefix+verb utterance when the “go” signal was presented. One TMS pulse would fire during each trial. This experiment had eight pulse timings– 400ms, 4100ms, 5180ms, 5260ms, 5340ms, 5420ms, 5500ms, 5580ms (each represented by the stimulation symbol)–as well as a null pulse condition.

The second experiment increased the preparation time, compared with the first experiment, in order to distribute the cognitive load over time. Each trial was 8800ms long. The participants were instructed to silently memorise a verb prior to the speech signal, to allow for preparation (see Fig 4 for an example).

#### 2.5.3 –Third experiment–delayed speech with pacing

**Fig 5:**
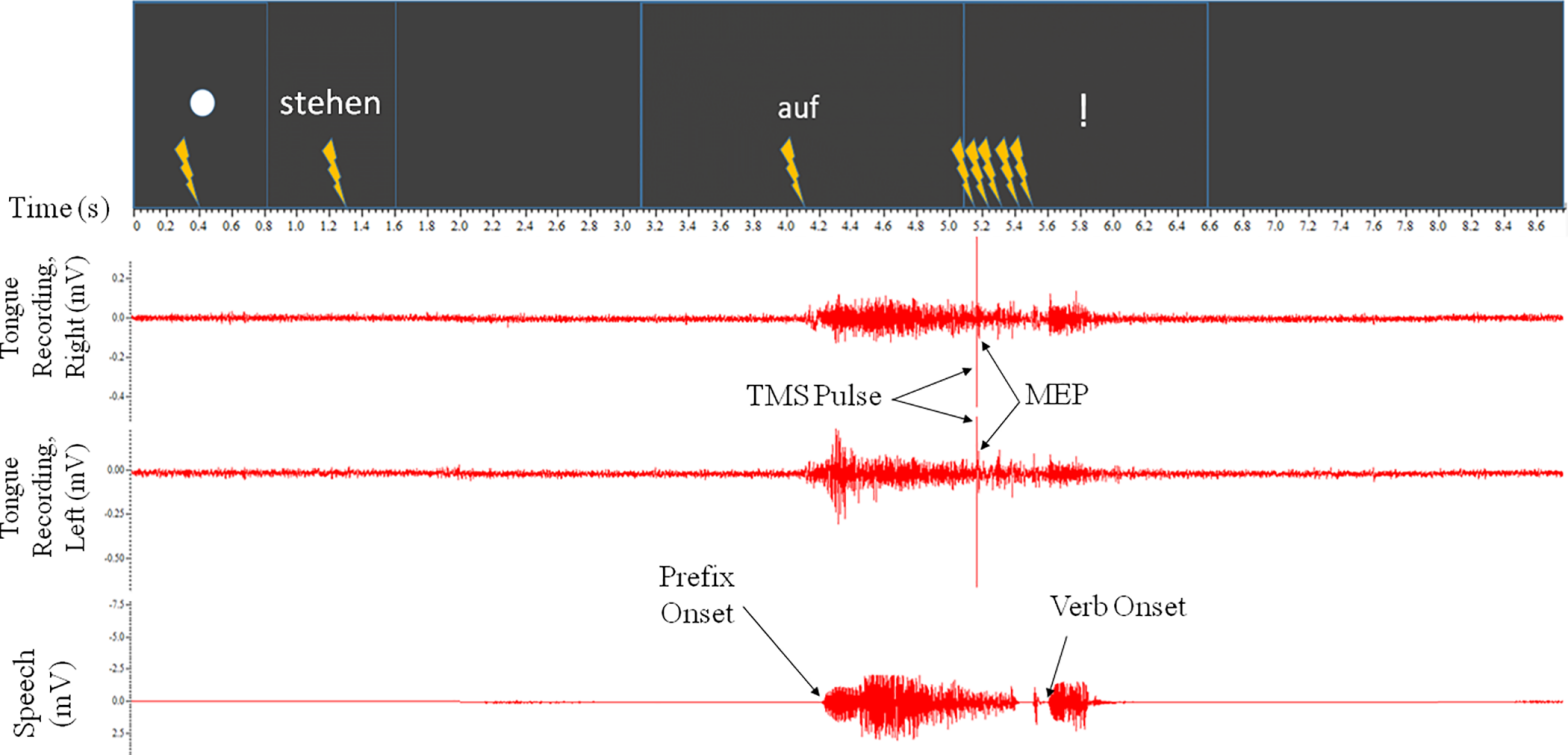
Example trial from Experiment 3. From the participant’s perspective, each trial began with the “alertness” signal of the white circle for 800ms. This was followed by a verb for 800ms, then a blank interval of 1500ms. The “auf” signal was then presented for 2000ms, followed by the “go” signal of a white exclamation mark. This was followed by a blank screen for 2200ms, for a total trial length of 8800ms. Participants spoke and held the prefix during the “auf” signal and transitioned into the verb at the “go” signal. One TMS pulse would fire during each trial. This experiment had eight pulse timings– 400ms, 1300ms, 4100ms, 5180ms, 5260ms, 5340ms, 5420ms, 5500ms (each represented by the stimulation symbol)–as well as a null pulse condition.

The third experiment replicated the previous design [24]. As in experiment two, each trial was 8800ms long and began with silent memorisation of the verb (see Fig 5). Participants were instructed to pronounce the “auf” for the full duration of the presentation, prolonging the labio-dental fricative (“auffffff”), before transitioning into the remembered verb (1500ms).

### 2.6.0 –Experimental design

Each participant was tested in a single session of TMS, with left hemispheric stimulation in all cases. In each session, we began with baseline assessments of the resting motor threshold and input-output curve. This was followed by a familiarisation period, where the participants were asked to perform 5 trials of the speech task with the mouthpiece in the correct position but without delivery of TMS pulses; if the participant was uncertain, we performed another 5 trials. In the first experiment, we conducted 120 trials which were split into four blocks of 30; due to practical laboratory constraints, the second and third experiments consisted of 108 Trials which were split into four blocks of 27 trials. Pauses between blocks lasted 5 minutes. A single TMS pulse was delivered per trial.

### 2.7.0 –Data analysis

We analysed the data using a custom-written EMG-Browser in Signal (Signal-2.16) and then Matlab (2015b). In Signal, we visually inspected the acoustic speech waveforms and determined the speech onset times of the prefix and verb using script markers which were confirmed by manual precision. Similarly, we visually examined the EMG signals from all recordings, used scripts to mark simple min/max values in the time window 10-30ms after the pulse artefact, then manually corrected for precision. Trials with extreme artefacts of either the EMG or the audio signal were removed. We then stored the millivolt difference in the peak to peak amplitudes in both left and right channels, which were extracted separately for each experiment, for the contralateral and ipsilateral projection in the left hemisphere.

To account for the differences in reaction and hesitation between trials and participants, we cut and re-zeroed all data to the moment of speech onset–to the prefix in experiments 1–2 and to verb onset in experiment 3. Trials were then removed by software analysis based on signal consistency and timing of stimulation–trials were excluded if the recording was too noisy, if there was no pulse artefact or if no pulse could be identified. All invalid recordings were excluded from the respective analyses.

### 2.8.0 –Statistical analyses

#### 2.8.1 –Statistical analyses–peak-to-peak MEP amplitude

To examine the relationship between pre-speech onset and MEP amplitude, we normalised the speech trials to the mean of non-speech trials then conducted a series of hierarchical multiple regression analyses (HMRAs). To control for individual variation, we first calculated the mean of the MEPs recorded during the non-speech tasks. For each experiment and each individual, we pooled the data from pulses 1 & 2 in order to maximise power (t-tests during the pilot study indicated that the two pulses were not significantly different in each experiment). We then divided each MEP response for pulses 3–9 by that participant’s mean baseline score for that experiment. Thus, MEP values represent a ratio score.

As the MEPs were centred on speech, the density of the data reduced rapidly beyond 600ms before speech. To avoid bias from outliers, the data was cut. We used a 500ms window, ending at 40ms after onset of speech. Additionally, extreme MEPs (relative to baseline) were capped at 10x baseline.

To test the primary hypotheses, we conducted HMRAs with the baseline normalised MEPs as the dependent variable and two blocks of predictors:

1. onset of speech–prefix (exp. 1 and exp. 2) or verb (exp. 3), and
2. group (control vs stuttering), side of recording (left/right tongue) and group-TSO interaction (group * MEP-TSO).

In accordance with standard techniques [38], we tested the various statistical assumptions before running the HMRAs for each experiment. For the assumption of normality of distribution, we used stem-and-leaf plots and we observed a small deviation from normality (skewed low), however this was deemed to be within the limits of robustness for the analysis chosen. Upon secondary inspection of the normal probability plot of standardised residuals and the scatterplot of standardised residuals against standardised predicted values, it appeared that the data was more tightly grouped around the mean but that the assumptions of normality, linearity and homoscedasticity of residuals were met. Additionally, all three experiments exhibited high tolerances for all predictors in the final regression model, which indicated that multicollinearity would not be a cause for concern in our HMRA.

As the outcome measures of the HMRAs are ratio data (raw score / mean baseline), the t-statistic of the regression constant was recalculated against a null hypothesis of 1 (no difference between raw score and baseline) instead of the standard null hypothesis of 0. The other variables were not affected.

To test the secondary hypotheses, we used the same HMRA as above, but substituted ‘percentage of stuttered syllables’ (%SS) in place of ‘group’ and generated a new interaction term between %SS and MEP-TSO.

To compare between the experiments (hypothesis 4), we conducted unpaired t-test comparisons of the distributions in each experiment. We used an online calculator (GraphPad) to compare the unstandardised coefficients (B), standard errors and sample size (n). All blocks of the regression output were compared (constant, speech onset interval, group/%SS and the interaction effect of group/%SS and MEP-TSO). To control for multiple analyses within each experiment, we used Bonferroni correction (sig. at p = ·0125).

#### 2.8.2 –Statistical analyses–reaction time

In keeping with the previous study [24], we analysed reaction times to all three experiments; we considered group membership, state of stimulation and trial number as factors in the reaction time analysis. Each of these factors were entered stepwise into an HMRA, to test the hypothesis that there would be a difference in response timing as a function of the TMS to Go-signal interval, both overall (Hyp3) and between groups (Hyp4).

As above with MEP analyses, we tested the statistical assumptions in accordance with standard techniques [38]; similarly, the minor deviations from normality were deemed acceptable.

### 3.0.0 –Results

#### 3.1.1 –Results–peak to peak MEP amplitude

In total, we ran six HMRA regression analyses of MEP amplitude–one ‘group’ and one with percentage of stuttered syllables (%SS) per experiment–as well as two sets of post-hoc t-test comparisons for the ‘group’ results from Experiment 1 and each of the other two experiments. All three ‘group’ level analyses were significant at both steps of each regression (see Tables 2–4), with their respective post-hoc analyses (see Tables 5 & 6), as were the three %SS results (see Tables 7–9).

**Table 2:** 

		Unstandardized Coefficients		Standardized Coefficients	t	Sig.	95% CI for B	
Model		B	Std. Error	Beta			Lower Bound	Upper Bound
1	(Constant)	1·823	0·040		20·575	<0·001	1·744	1·902
	TPO	2·347	0·151	0·241	15·504	<0·001	2·050	2·644
2	(Constant)	2·190	0·083		14·337	<0·001	2·027	2·352
	TPO	3·155	0·216	0·323	14·634	<0·001	2·732	3·578
	Side	-0·034	0·040	-0·013	-0·840	0·401	-0·112	0·045
	Group	-0·607	0·080	-0·234	-7·598	<0·001	-0·764	-0·451
	Group-TPO Interaction	-1·585	0·301	-0·183	-5·274	<0·001	-2·174	-0·996

**Hierarchical multiple regression for Experiment 1.** B = unstandardized coefficient. TPO = Time before Prefix Onset. Side = Side of tongue recording. Group was binary (0 = control, 1 = AWS). The constant of the regression was tested against an H0 of B = 1, instead of the normal B = 0.

**Table 3:** 

		Unstandardized Coefficients		Standardized Coefficients	t	Sig.	95% CI for B	
Model		B	Std. Error	Beta			Lower Bound	Upper Bound
1	(Constant)	1·407	0·040		10·175	<0·001	1·330	1·485
	TPO	1·679	0·150	0·187	11·158	<0·001	1·384	1·974
2	(Constant)	1·479	0·081		5·9136	<0·001	1·321	1·637
	TPO	2·175	0·215	0·242	10·096	<0·001	1·752	2·597
	Side	0·058	0·038	0·026	1·536	0·125	-0·016	0·132
	Group	-0·302	0·079	-0·134	-3·817	<0·001	-0·457	-0·147
	Group-TPO Interaction	-0·943	0·301	-0·121	-3·136	0·002	-1·532	-0·353

**Hierarchical multiple regression for Experiment 2.** B = unstandardized coefficient. TPO = Time before Prefix Onset. Side = Side of tongue recording. Group was binary (0 = control, 1 = AWS). The constant of the regression was tested against an H0 of B = 1, instead of the normal B = 0.

**Table 4:** 

		Unstandardized Coefficients		Standardized Coefficients	t	Sig.	95% CI for B	
							Lower Bound	Upper Bound
Model		B	Std. Error	Beta				
1	(Constant)	1·537	0·049		10·959	<0·001	1·440	1·633
	TVO	0·562	0·210	0·052	2·671	0·008	0·149	0·974
2	(Constant)	1·573	0·106		5·406	<0·001	1·366	1·781
	TVO	0·860	0·292	0·079	2·941	0·003	0·286	1·433
	Side	0·080	0·053	0·029	1·509	0·131	-0·024	0·183
	Group	-0·305	0·098	-0·111	-3·110	0·002	-0·498	-0·113
	Group-TVO Interaction	-0·526	0·420	-0·049	-1·252	0·211	-1·351	0·298

**Hierarchical multiple regression for Experiment 3.** B = unstandardized coefficient. TVO = Time before Verb Onset. Side = Side of tongue recording. Group was binary (0 = control, 1 = AWS). The constant of the regression was tested against an H0 of B = 1, instead of the normal B = 0.

**Table 5:** 

	Exp1-Exp2	SE of Diff	CI Low	CI High	t	df	Sig.
Constant	0·711	0·117	0·48184	0·94016	6·0959	7357	<0·001
TPO	0·98	0·306	0·37857	1·58143	3·2014	7357	0·0014
Group	-0·305	0·113	-0·52699	-0·08301	2·6994	7357	0·007
Interaction	-0·642	0·427	-1·4818	0·1978	1·502	7357	0·1331

**Secondary Data Analysis–Between experiments comparison for Group Difference between experiments 1 and 2.** TPO = Time before Prefix Onset. Group was binary, with 0 = controls and 1 = AWS. Interaction represents the Group*TPO interaction.

**Table 6:** 

	Exp1-Exp3	SE of Diff	CI Low	CI High	t	df	Sig.
Constant	0·617	0·133	0·35519	0·87881	4·6301	6592	<0·001
TPO/TVO	2·295	0·356	1·59612	2·99388	6·4518	6592	<0·001
Group	-0·302	0·126	-0·54995	-0·05405	2·393	6592	0·0167
Interaction	-1·059	0·503	-2·04746	-0·07054	2·1049	6592	0·0353

**Secondary Data Analysis–Between experiments comparison for Group Difference between experiments 1 and 3.** TPO/TVO = Time before Prefix/Verb Onset. Group was binary, with 0 = controls and 1 = AWS. Interaction represents the Group*TPO/TVO interaction.

**Table 7:** 

		Unstandardized Coefficients		Standardized Coefficients	t	Sig.	95% CI for B	
							Lower Bound	Upper Bound
Model		B	Std. Error	Beta				
1	(Constant)	1·823	0·040		20·575	<0·001	1·744	1·902
	TPO	2·347	0·151	0·241	15·504	<0·001	2·050	2·644
2	(Constant)	1·984	0·077		12·779	<0·001	1·833	2·136
	TPO	2·696	0·179	0·276	15·057	<0·001	2·345	3·047
	Side	-0·033	0·040	-0·013	-0·827	0·408	-0·112	0·045
	%SS	-1·808	0·436	-0·130	-4·147	<0·001	-2·663	-0·953
	%SS-TPO Interaction	-5·873	1·675	-0·114	-3·506	<0·001	-9·158	-2·589

**Hierarchical multiple regression for Experiment 1 using Percentage of Stuttered Syllables.** B = unstandardized coefficients. TPO = Time before Prefix Onset. Side = Side of tongue recording. %SS = Percentage of Stuttered Syllables. %SS was a continuous variable including all previously included data from both groups. The constant of the regression was tested against an H0 of B = 1, instead of the normal B = 0.

**Table 8:** 

		Unstandardized Coefficients		Standardized Coefficients	t	Sig.	95% CI for B	
							Lower Bound	Upper Bound
Model		B	Std. Error	Beta				
1	(Constant)	1·407	0·040		10·175	<0·001	1·330	1·485
	TPO	1·679	0·150	0·187	11·158	<0·001	1·384	1·974
2	(Constant)	1·346	0·075		4·613	<0·001	1·199	1·492
	TPO	1·661	0·182	0·185	9·146	<0·001	1·305	2·017
	Side	0·058	0·038	0·026	1·538	0·124	-0·016	0·132
	%SS	-0·385	0·425	-0·032	-0·905	0·365	-1·218	0·448
	%SS-TPO Interaction	0·510	1·582	0·012	0·322	0·747	-2·593	3·612

**Hierarchical multiple regression for Experiment 2 using Percentage of Stuttered Syllables.** B = unstandardized coefficients. TPO = Time before Prefix Onset. Side = Side of tongue recording. %SS = Percentage of Stuttered Syllables. %SS was a continuous variable including all previously included data from both groups. The constant of the regression was tested against an H0 of B = 1, instead of the normal B = 0.

**Table 9:** 

		Unstandardized Coefficients		Standardized Coefficients	t	Sig.	95% CI for B	
							Lower Bound	Upper Bound
Model		B	Std. Error	Beta				
1	(Constant)	1·537	0·049		10·959	<0·001	1·440	1·633
	TVO	0·562	0·210	0·052	2·671	0·008	0·149	0·974
2	(Constant)	1·536	0·099		5·414	<0·001	1·343	1·730
	TVO	1·067	0·244	0·098	4·371	<0·001	0·588	1·546
	Side	0·079	0·053	0·029	1·505	0·132	-0·024	0·183
	%SS	-2·141	0·561	-0·129	-3·813	<0·001	-3·242	-1·040
	%SS-TVO Interaction	-9·291	2·329	-0·141	-3·990	<0·001	-13·858	-4·725

**Hierarchical multiple regression for Experiment 3 using Percentage of Stuttered Syllables.** B = unstandardized coefficient. TPO = Time before Prefix Onset. Side = Side of tongue recording. %SS = Percentage of Stuttered Syllables. %SS was a continuous variable including all previously included data from both groups. The constant of the regression was tested against an H0 of B = 1, instead of the normal B = 0.

**Fig 6:**
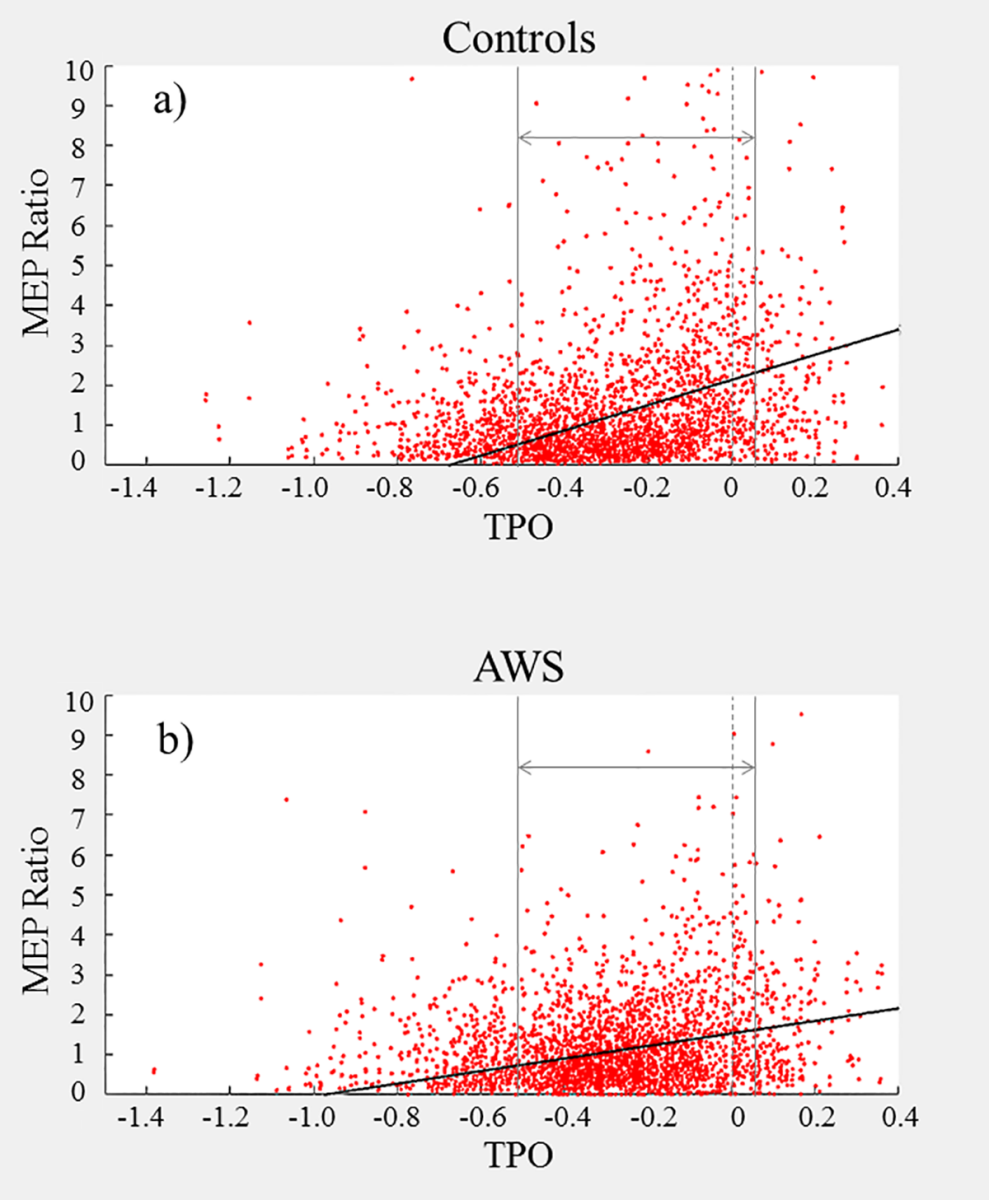
**a-b. Experiment 1 Pulse Interval–MEP Peak to Peak Amplitude relative to prefix onset for Controls (a) and AWS (b).** Each point represents one trial. Prefix onset for each trial is marked by the dashed vertical bar at TPO = 0. The outer vertical bars denote the 500ms time window chosen for analysis. The diagonal line represents the linear regression predicted by the model applied within the selected window.

**Fig 7:**
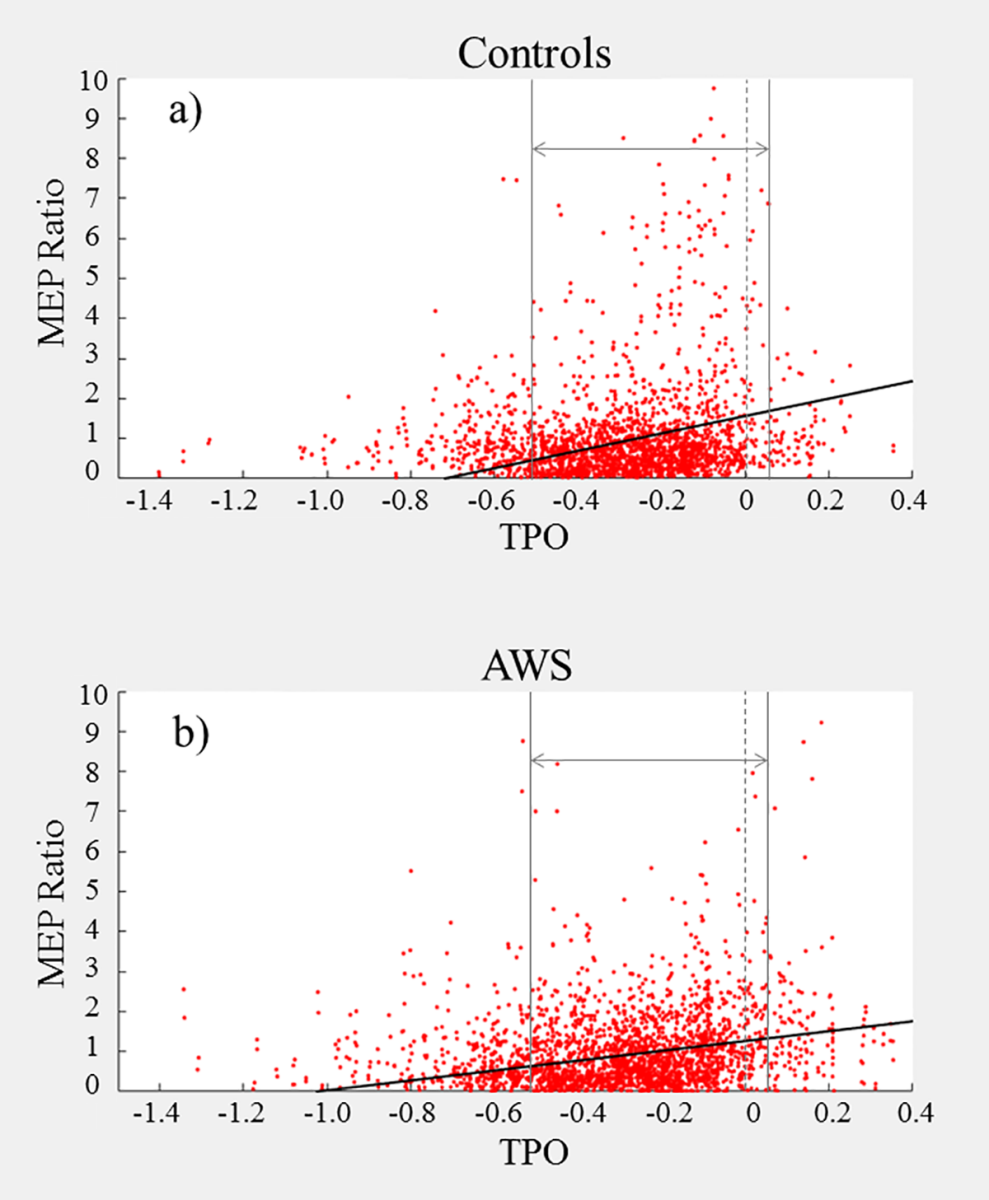
**a-b. Experiment 2 Pulse Interval–MEP Peak to Peak Amplitude relative to prefix onset for Controls (a) and AWS (b).** Each point represents one trial. Prefix onset for each trial is marked by the dashed vertical bar at TPO = 0. The outer vertical bars denote the 500ms time window chosen for analysis. The diagonal line represents the linear regression predicted by the model applied within the selected window.

**Fig 8:**
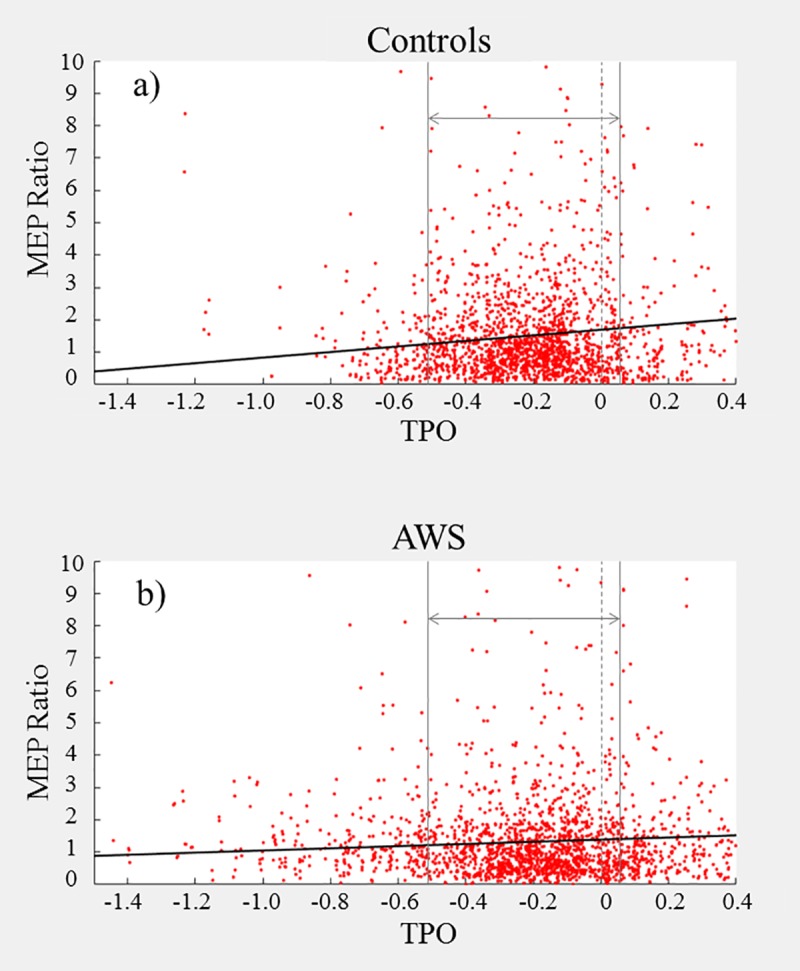
**a-b. Experiment 3 Pulse Interval–MEP Peak to Peak Amplitude relative to verb onset for Controls (a) and AWS (b).** Each point represents one trial. Verb onset for each trial is marked by the dashed vertical bar at TPO = 0. The outer vertical bars denote the 500ms time window chosen for analysis. The diagonal line represents the linear regression predicted by the model applied within the selected window.

Our broad findings indicate that AWS exhibit reduced MEPs than ANS, both overall and over time. The difference was most pronounced in the first experiment, where the two groups diverged early (see Fig 6). The second experiment was similar, but with reduced responses by both groups relative to the first experiment and marginally later divergence (see Fig 7). The third experiment exhibited a reduced peak level, relative to the first, but the overall MEPs maintained a moderately high level relative to baseline throughout the experiment (see Fig 8); this is likely due to the design of the pacing condition. It should be noted that all three experiments characterised a small effect size according to *R*^*2*^ (see supplementary information).

**Fig 9:**
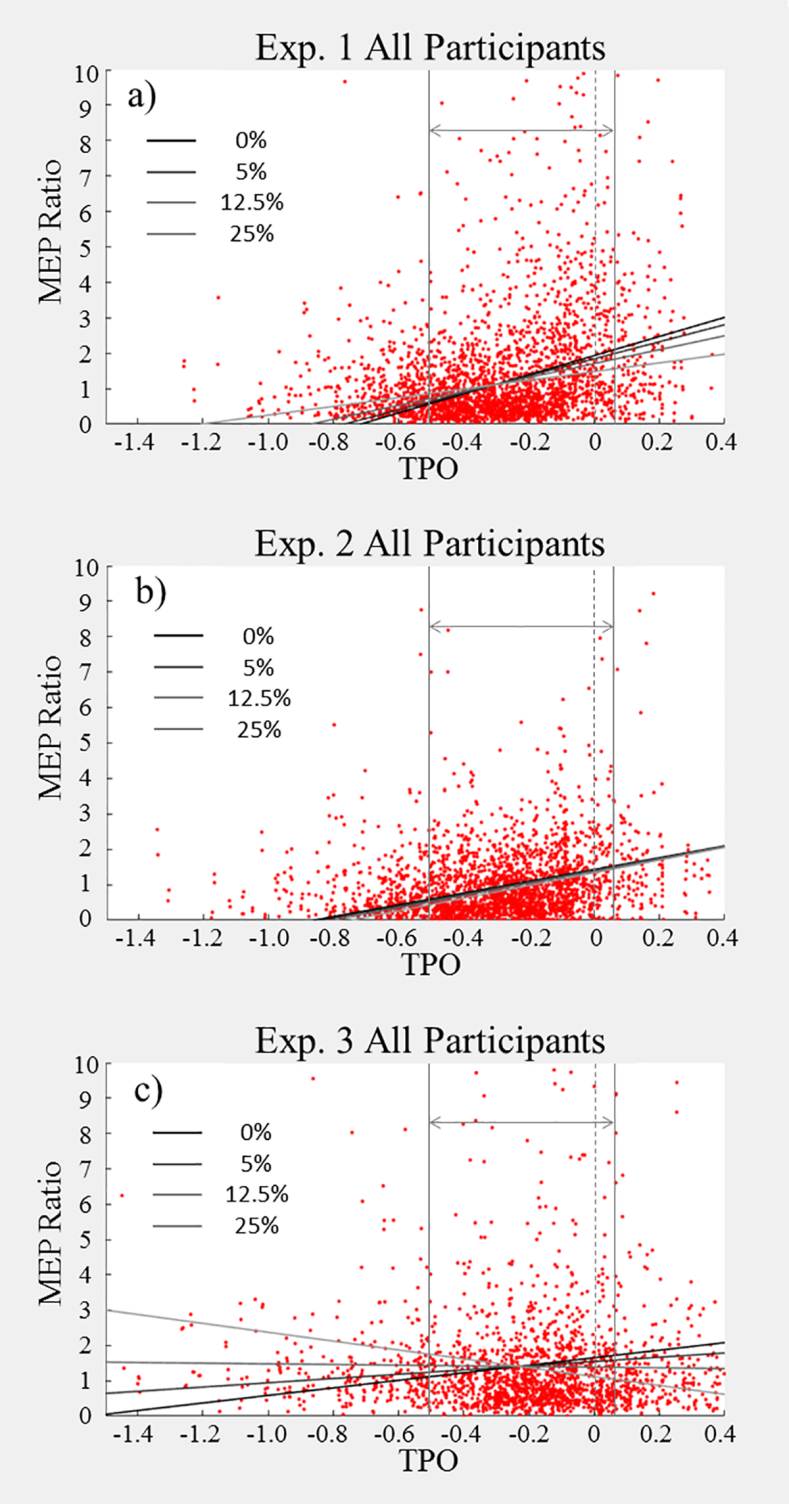
**a-c Linear Regression predictions based on stuttering severity (%SS) with respect to time before speech.** Each point represents one trial. Each figure includes all data for that experiment, irrespective of group. Prefix onset (Figs 9a & 9b) and verb onset (Fig 9c) for each trial is marked by the dashed vertical bars at TPO = 0. The outer vertical bars denote the 500ms time window chosen for analysis. The diagonal line represents the linear regression predicted by the model applied within the selected window, differentiating between levels of stuttering (none, low, medium and high).

Our secondary %SS findings supported the primary between-groups findings for the first and third experiments, but the *delayed speech without pacing* experiment 2 did not elicit a significant effect of stuttering. Fig 9A–9C give an overview of these findings, which will be explained in more detail below.

#### 3.1.2 –Results–specific MEP findings by experiment

As noted in the methods, all regression constants were recalculated against a null hypothesis of 1 (no significantly different ratio). All recalculated regression constants were significant for all experiments (see the respective Tables 2–7); this indicates that, on average, the MEPs over the trial window was greater than the MEPs during the non-speech baseline period.

For the first experiment (*immediate* speech), 3916 trials were included in the analysis (see Table 2, with additional model information in the supporting information file under S2 Table). The first block of the regression indicated a positive correlation between pre-speech interval and MEP response; this effect increased when accounting for all other factors. There were also two negative correlations: one between ‘MEP’ and ‘group’ and the other between ‘MEP’ and the ‘group to MEP-TSO’ interaction. Due to dummy coding, this negative correlation indicates that AWS exhibited a reduced average MEP and a lesser increase in MEP closer to speech onset compared with controls.

For the second experiment (*delayed without pacing*), 3443 trials were included in the analysis (see Table 3, with additional model information in the supporting information file under S3 Table). As in experiment 1, there was a positive correlation between MEP amplitude and pre-speech interval, as well as negative correlations for both ‘group’ and the ‘group to MEP-TSO’ interaction.

For the third experiment (*delayed with pacing*), 2678 trials were included in the analysis (see Table 4, with additional model information in the supporting information file under S4 Table). As before, there was a positive correlation with the regression constant and with the pre-speech interval, as well as a negative correlation with ‘group’; however, the ‘group to MEP-TSO’ interaction effect was not significant.

#### 3.1.3 –Results–post-hoc analyses of the primary regressions

In post-hoc analyses, we compared the regression outputs of the first experiment with the other two experiments. Overall, the second experiment elicited lower MEPs than the first in all comparisons except the ‘group to MEP-TSO’ interaction (see Table 5 for comparisons and S1 Text Appendix for more information). Similarly, the third experiment elicited lower MEPs than the first–the constant and pre-speech interval were significantly smaller than in the first experiment, while group and the ‘group to MEP-TSO’ interaction were marginally non-significant after Bonferroni correction (see Table 6 for comparisons and S2 Text Appendix for more information).

#### 3.2.0 –Results–secondary regression analyses

As above, the regressions for %SS had the same participant numbers and the same first blocks in each analysis (see Tables 7–9). The unique contribution of the second block of the regression was significant for all three analyses, as indicated by the significant F Change (see supplementary S5–S7 Tables).

Stuttering was a significant predictor in both the first and third experiments, as was the ‘group to MEP-TSO’ interaction effect of group and pre-speech MEP time. As both were positively coded, this indicates that better fluency correlates with higher MEPs and this correlation increases faster with greater fluency. In experiment two, the only significant variable in the second block was the MEP-TSO.

### 3.3.0 –Results–reaction time

In total, we ran four HMRAs: three for *Prefix Reaction Time* (exp. 1–3, Tables 10A–10C) and one for *Verb Reaction Time* (exp. 3, Table 10D). Overall, AWS were slower than ANS in the first and third experiment but not the second. The responses also got faster over the course of experiments 1 and 3 irrespective of group, but not in experiment 2 –this indicated that the participants were learning the precise trial timing. Additionally, responses were slower among all participants when pre-speech pulse intervals were smaller; this implies a level of distraction from the pulse.

**Table 10:** 

n = 3623	Auf RT	Group	Pulse	Trial	n = 3211	Auf RT	Group	Pulse	Trial
Auf RT	-	-0·044**	0·185***	-0·078***	Auf RT	-	-0·018	0·135***	0·022
(Sig.)		0·004	(<0·001)	(<0·001)	(Sig.)		0·156	(<0·001)	0·106
Group		-	-0·01	0·001	Group		-	-0·001	-0·007
(Sig.)			0·275	0·48	(Sig.)			0·478	0·346
State			-	0·052**	State			-	0·016
(Sig.)				(<0·001)	(Sig.)				0·184
Trial				-	Trial				-
**10a**					**10b**				
n = 3172	Auf RT	Group	Pulse	Trial	n = 3172	Verb RT	Group	Pulse	Trial
Auf RT	-	-0·014	0·002	-0·068***	Verb RT	-	-0·040*	0·030*	-0·032*
(Sig.)		0·221	0·445	(<0·001)	(Sig.)		0·013	0·043	0·036
Group		-	-0·001	-0·015	Group		-	-0·001	-0·015
(Sig.)			0·476	0·192	(Sig.)			0·476	0·192
State			-	-0·012	State			-	-0·012
(Sig.)				0·245	(Sig.)				0·245
Trial				-	Trial				-
**10c**					**10d**				

**Hierarchical multiple regression analyses for reaction time to prefix in each experiment (10a-c) and verb in experiment 3 (10d).** Correlation values for each pair, with significances shown in parentheses (* = p < ·05, ** = p < ·01, *** = p < ·001). Negative correlation values represent reductions in reaction time, thereby a faster response by the participant. The significant values of Trial in experiments 1 and 3 indicate that the participants were learning the precise trial timing. The Trial-State interaction in Table 10A is discussed in S3 Text Appendix.

## 4.1.1 –Discussion

Broadly speaking, our results demonstrate that AWS exhibit reduced facilitation compared with ANS prior to speech onset, but this difference is not consistent across conditions. This difference is also not simply a difference in magnitude of facilitation–using between-groups and between-experiments comparisons, our findings indicate that the discrepancy was greatest during our *immediate* condition, but also that the facilitation within both groups diminished under increased speech disturbance. These findings will be discussed in more detail below.

### 4.1.2 –Note–Facilitation or disinhibition?

It should be noted that changes in MEP response can be due to one of two mechanisms:

1. increased facilitation–up-regulation in cortical positive feedback systems, or
2. reduced inhibition–down-regulation in negative feedback systems.

In our experiments, it is unclear whether increases in MEP are a result of increased facilitation or reduced inhibition. For simplicity, we use ‘MEP Facilitation’ to refer to our findings; however, it is possible also that the underlying cause is disinhibitory.

### 4.2.1 –MEP facilitation increases prior to speech

The regulation of cortical excitability forms the basis of motor action sequences, underlying the planning and execution behind smooth coordinated motion–in the moments before movement onset, the neural activity spikes. Our data demonstrates that the same holds true for speech motor activity–supporting our first hypothesis–and the positive ‘TPO/TVO’ correlation values in each experiment represent the magnitude of this relationship.

### 4.3.0 –Overall MEP facilitation is reduced in AWS

As stuttering is a speech movement disorder associated with reduced control particularly at movement onset, we hypothesised that AWS would exhibit reduced MEP facilitation, compared with ANS. Again, our results supported this across all three experiments–the significant negative correlation of ‘group’ suggests that AWS have a significantly diminished overall response than they had at baseline, compared with ANS.

This pattern of increasing MEP Facilitation prior to initiation of motor tasks is analagous to the Bereitschaftspotential (BP). BP research has identified two distinct stages–early (BP1) and late (BP2), beginning around 1200ms and 500ms before movement initiation respectively. It is believed that BP1 originates in the anterior supplementary motor area (SMA) and the pre-SMA, while BP2 probably arises in both the SMA and the contralateral motor cortex (Colebatch, 2007). Many movement disorders are associated with a disturbed BP; for example, the BP is absent in disorders like Parkinson’s disease (e.g. [39, 40] and overactive in tic disorders (e.g. [41]) when looking at self-triggered movements. Given our findings of reduced facilitation in AWS, and that of reduced contralateral facilitation in previous research [24], we anticipate a link between MEP and BP in AWS which should be investigated in future research.

### 4.4.0 –AWS exhibit reduced growth in MEPs as a function of time before speech

As demonstrated by the negative ‘group to MEP-TSO’ interaction effect, AWS exhibited reduced MEP facilitation as a function of time compared with ANS. However, unlike the two findings above, the ‘group to MEP-TSO’ interaction effect was significant in the *immediate* condition and the *delayed speech without pacing* condition, but not the *delayed with pacing* condition. Note that in the pacing condition, the MEP was recorded during the verb after holding the prolonged prefix. In combination with the significant between-groups finding above, this suggests that AWS have a reduced dynamic range during pre-speech facilitation. As suggested by the Bereitschaftspotential model, one explanation is that AWS lack the late stage rapid facilitation immediately before speech onset in normal speech. However, in highly disrupted speech, the difference disappears. This will be addressed in combination with all three experiments below.

### 4.5.0 –Stuttering is not simply derived from a reduction in magnitude of facilitation

It has been previously suggested that there may be a link between stuttering and cognitive load [25] or working memory capacity [26, 42]; however, there is no broader agreement on the nature of the link and its role in AWS. This is likely due to the many different methods used to assess the construct. We examined this controversy by comparing between our three experiments. It can be considered that the spontaneous production of speech in our *immediate* condition represented the highest demand on cognitive load (e.g. [25]); alternatively, it can be interpreted that the drawn out *delayed with pacing* condition placed additional working memory demands from both the planning of the movement and the external disruptions to speech (e.g. [26]).

The technique of artificially drawing out words, as in the third experiment, has been employed in many stuttering therapies to reduce the severity of stuttering. For this paper, we therefore anticipated that such increased speech interference and preparation time, which improve speech fluency in therapy, would manifest in improvements in MEP facilitation. Thus our fourth hypothesis anticipated that the between-groups difference would be smallest in the pacing experiment (exp. 3), as AWS facilitation approached ANS levels, and greatest in the *immediate* experiment (exp. 1).

On the whole, our fourth hypothesis proved correct–the increased timing, which is provided by drawing out the utterance, reduces the concurrent complexity for AWS and results in a trend towards ANS performance. However, our group differences were not linear across the three experiments. Although we did find that the *immediate* experiment (exp. 1) elicited a greater difference in MEPs between groups than either of the other two (see Tables 5A–6A), there was no difference between the second and third experiments. Rather, in combination with the other findings above, it appears that ANS exhibited a reduced facilitation over time, while AWS exhibited average magnitude (the constant) but no change in facilitation over time. In combination with the previous study [24], where they found that ANS have increased left hemispheric facilitation prior to speech but AWS do not, this implies the existence of a speech specialised programme of facilitation in ANS. This additionally supports the suggestion, by Bosshardt [25], of a reduced level of “modularization” in the neural subsystems involved in speech planning in AWS. Further, it suggests that this modularization is speech specific. Specifically, under normal everyday speech conditions, ANS have a specialisation for speech preparation that does not generalise to non-speech mouth movement actions; conversely, AWS do not have this specialisation for speech coordination, and must rely on their general movement preparation sequences for all forms of speech. However, this remains speculative at this stage–further research incorporating undisturbed speech is needed.

### 4.6.0 –Reduced MEP facilitation in AWS is not simply a result of stutter-like delays

One might argue that these group differences in MEP facilitation may be solely an artefact of experimental design–i.e. as our facilitation data was zeroed to the moment of speech onset, reaction time delays due to stuttering events could reduce the average AWS results. This can be refuted by a few arguments. Firstly, there were no stutter-like events during speech noted by the experimenters and double-checked with the audio recording; this is possibly a result of having a large mouthpiece impeding speech (e.g. [27]. Secondly, a visual inspection of the facilitation graphs (see Figs 6–8) shows that the main bulk of trials in both groups falls in the same time window–if there were a stutter-based delay, we would expect to see a longer, thinner band of data for AWS of at least a few hundred ms.

The third argument addresses the first of our secondary hypotheses–that reaction time would differ between groups, with AWS responding slower across all three experiments, even after accounting for the distraction of pulse timing and learning. As shown in Table 10, AWS responded approximately 40ms slower than ANS in both the first experiment and the verb component of the third experiment, while the second experiment and the prefix component of the third experiment were not significantly different; conversely, the MEP group difference was significant for all three. Although the trend suggested slower reaction times for AWS in all components on average, thus supporting the hypothesis of slower reaction times in AWS, the differences were not uniform. This suggests partial independence between the MEP and reaction time.

The non-uniform reaction times also lend additional support to the possibility of a speech movement specialisation in ANS, which is missing in AWS. While the drawn out speech in the two prepared speech conditions reduces the working memory overload in AWS, it concurrently abstracts away from standard speech for which ANS are specialised.

### 4.7.0 –Severity of stuttering or group analysis

As with many developmental disorders, stuttering is diagnosed categorically. However, our second secondary hypothesis suggests that stuttering may fall on a continuum, whereby severity of stuttering (and possibly type–e.g. more blockages, fewer elongations) may better illustrate the question of pre-speech preparatory facilitation than a simple binary diagnosis. For simplicity, we used only the total Percentage of Stuttered Syllables (%SS), but not the symptom type–that would deserve a full paper itself. Using this method, we found similar results for both input methods in the *immediate* speech experiment–the simple %SS and the ‘%SS to MEP-TSO’ interaction effects were both present and stronger than in the between-groups analysis. For the other two experimental conditions, the findings were slightly different.

Firstly, the *delayed speech with pacing* produced better results than in the between-groups analysis–in particular, we found a strong ‘group to MEP-TSO’ interaction effect that was not demonstrated in the groups analysis. This strong negative effect predicts that in individuals with high severity, MEP facilitation would already be on the decline after the prefix before the verb is actually spoken (see Fig 9C). Whether this holds true in reality requires further investigation.

The *delayed without pacing* condition represented the biggest deviation from our between-groups analysis–in this analysis, the only significant component of the regression was time. Upon visual inspection of the individual data, the likely cause for this was the fact that the very mild and mild AWS had elicited unexpectedly low MEPs. There was the appearance of a linear effect of %SS in the moderate-very severe AWS, but the very mild participants had performed equivalently to the very severe in this experimental condition. This may be due to small *n* effects; however this requires further investigation. As a result, however, we recommend against using %SS in place of group difference unless it is a dedicated examination of this analytical design.

## 5.0.0 –Conclusion–external speech interference affects AWS and ANS differently

When taken all together, our data suggests that ANS and AWS handle external interference with their speech preparation in functionally distinct manners. It suggests the existence of a speech specific preparation programme in ANS that is lacking in AWS. This is supported by the increase in late stage facilitation in *immediate* speech in ANS, analogous to the late phase Bereitschaftspotential. Additionally, as there were very few stuttering events during the experiment even among participants classified as severe, it supports the idea that stuttering belies a chronic neurophysiological aberration that extends beyond the individual utterance [24].

Our data also suggests the mechanisms underlying the efficacy of moderate external interference in treating stuttering, such as is employed in fluency shaping (e.g. [43–45]–both the reduction in working memory overload and the improvement in preparatory facilitation. Future research to investigate these mechanisms could incorporate active cortical stimulation, such as transcranial direct or alternating current stimulation (tDCS/tACS, see [46]. When employed to up-regulate pre-speech facilitation, it may permit greater preparation in spontaneous speech and improve therapy outcomes, as has been seen in stimulation studies with Parkinson’s disease (e.g. [40, 47].

Our data did not allow conclusions with regard to the use of severity as an indicator of the neurophysiological mechanisms. This may be due to a more complex and non-linear relationship between stuttering severity and motor facilitation–for example, it may be that individuals with mild stuttering have a weak specialisation for speech, one that is easily overwhelmed in our interference conditions; alternatively, it may be a quirk of analysis. A dedicated investigation, likely including the non-verbal concomitant symptoms of stuttering, is needed to identify the precise nature of this relationship in the future.

## Supporting information

S1 Table Individual stuttering demographics–Group was used for the main analyses. Percentage of stuttering was used for the supplementary analyses.
(DOCX)

S2 Table Model Summary for Experiment 1 –Additional statistical information pertaining to Table 2 in the text. (DOCX)

S3 Table Model Summary for Experiment 2 –Additional statistical information pertaining to Table 3 in the text. (DOCX)

S4 Table Model Summary for Experiment 3 –Additional statistical information pertaining to Table 4 in the text. (DOCX)

S5 Table Model Summary for Experiment 1 –Additional statistical information pertaining to Table 7 in the text. (DOCX)

S6 Table Model Summary for Experiment 2 –Additional statistical information pertaining to Table 8 in the text. (DOCX)

S7 Table Model Summary for Experiment 3 –Additional statistical information pertaining to Table 9 in the text. (DOCX)

S1 Text Appendix (to Table 5)–Specific statistical analysis of the comparison between experiments 1 and 2. The constant (*M* = 0.711, *SED* = 0.117, *p* < .001), pre-speech interval (*M* = 0.980, *SED* = 0.306, *p* < .01) and group (*M* = -0.305, *SED* = 0.113, *p* < .01) were all lower than in the *immediate* condition, even after Bonferroni correction (sig. p < 0.0125). The interaction did not differ significantly (*M* = -0.642, *SED* = 0.427, *p* = .133).
(DOCX)

S2 Text Appendix (to Table 6)–Specific statistical analysis of the comparison between experiments 1 and 3. The constant (*M* = 0.617, *SED* = 0.133, *p* < .001) and pre-speech interval (*M* = 2.295, *SED* = 0.356, *p* < .001) were significantly smaller than in the first experiment, while group (*M* = -0.302, *SED* = 0.126, *p* = .0167) and the group-interval interaction (*M* = -1.059, *SED* = 0.503, *p* = .035) were marginally non-significant after Bonferroni correction (sig. p < 0.0125)
(DOCX)

S3 Text Appendix (to Table 10A–10D)–Significance of reaction time with pulse condition and trial. As a side note, one significant value was unexpected–the correlation between State and Trial in the first experiment. This implies that there were more late stimulation states in the later trials, on average. However, due to the random order of presentation and the significant correlations between Reaction Time and both Pulse Condition and Trial, we felt that this did not impact the study adversely.
(DOCX)
