# Supplementary material for: Adults who stutter lack the specialised pre-speech facilitation found in non-stutterers
Source: PLoS One. 2018 Oct 10;13(10):e0202634. doi: 10.1371/journal.pone.0202634 (PMC6179203; doi:10.1371/journal.pone.0202634)
Supplement: S1 Table — Percentage of stuttering was used for the supplementary analyses. (DOCX) [file pone.0202634.s001.docx]

**S1 Table**

| ID | Group | Stuttering while  Reading (%) | Stutteirng while  Reading (SSI Score) | Stuttering in Spon.  Speech (%) | Stuttering in Spon.  Speech (SSI Score) | Stutter Duration  (SSI Score) | Concomitant Movements  (SSI Score) | SSI Overall Score | Severity  (SSI Rating) | Percentage of Stuttering | Age | Sex | Handedness Score | Education | Motor Threshold (%) | Family History  of Stuttering |
| --- | --- | --- | --- | --- | --- | --- | --- | --- | --- | --- | --- | --- | --- | --- | --- | --- |
| 1004 | Control | 0.81 | 0 | 0.20 | 0 | 4 | 1 | 5 | None | 0.50 | 30 | Male | 70 | 5 | 40 | No |
| 1005 | Control | 1.40 | 2 | 0.84 | 0 | 4 | 1 | 7 | None | 1.12 | 24 | Male | 100 | 4 | 47 | No |
| 1006 | Control | 1.00 | 2 | 0.00 | 0 | 4 | 0 | 6 | None | 0.50 | 27 | Male | 53.8 | 5 | 45 | No |
| 1007 | Control | 0.00 | 0 | 0.00 | 0 | 0 | 0 | 0 | None | 0.00 | 27 | Female | 86.7 | 5 | 42 | No |
| 1008 | Control | 1.40 | 2 | 0.00 | 0 | 4 | 3 | 9 | None | 0.70 | 21 | Male | 100 | 2 | 42 | No |
| 1009 | Control | 1.20 | 2 | 0.29 | 0 | 4 | 0 | 6 | None | 0.75 | 27 | Female | 100 | 5 | 41 | No |
| 1010 | Control | 0.20 | 0 | 0.00 | 0 | 2 | 0 | 2 | None | 0.10 | 22 | Female | 100 | 3 | 40 | No |
| 1011 | Control | 0.20 | 0 | 1.09 | 2 | 2 | 0 | 4 | None | 0.65 | 19 | Female | 100 | 2 | 45 | No |
| 1012 | Control | 1.00 | 2 | 1.43 | 2 | 2 | 1 | 7 | None | 1.22 | 22 | Female | 63 | 2 | 44 | No |
| 1013 | Control | 0.00 | 0 | 0.00 | 0 | 0 | 0 | 0 | None | 0.00 | 25 | Female | 70 | 4 | 44 | No |
| 1014 | Control | 0.20 | 0 | 0.00 | 0 | 2 | 0 | 2 | None | 0.10 | 22 | Female | 80 | 3 | 52 | No |
| 1015 | Control | 0.20 | 0 | 0.32 | 0 | 2 | 2 | 4 | None | 0.26 | 24 | Female | 100 | 4 | 46 | No |
| 1016 | Control | 1.60 | 2 | 0.68 | 0 | 2 | 0 | 4 | None | 1.14 | 20 | Male | 100 | 2 | 53 | No |
| 1017 | Control | 1.20 | 2 | 0.86 | 0 | 4 | 0 | 6 | None | 1.03 | 22 | Male | 100 | 4 | 53 | No |
| 1018 | Control | 0.60 | 0 | 1.87 | 2 | 2 | 0 | 4 | None | 1.24 | 30 | Male | 100 | 4 | 41 | No |
| 1019 | Control | 1.80 | 2 | 0.34 | 0 | 2 | 1 | 5 | None | 1.07 | 21 | Male | 77.8 | 4 | 44 | No |
| 1020 | Control | 0.20 | 0 | 0.00 | 0 | 2 | 0 | 2 | None | 0.10 | 25 | Male | 60 | 4 | 44 | No |
| 2010 | Stutter | - | - | - | - | - | - | 17 | V. Light | 3.10 | 43 | Male | 100 | 6 | 35 | Yes |
| 2011 | Stutter | 9.00 | 7 | 12.83 | 8 | 8 | 2 | 25 | Moderate | 10.92 | 22 | Male | 100 | 3 | 50 | No |
| 2012 | Stutter | 2.60 | 4 | 4.38 | 5 | 6 | 3 | 18 | Light | 3.49 | 27 | Male | 80 | 3 | 62 | No |
| 2013 | Stutter | 23.68 | 9 | 26.25 | 9 | 12 | 2 | 32 | Severe | 24.97 | 19 | Male | 100 | 2 | 43 | No |
| 2014 | Stutter | 4.48 | 5 | 4.13 | 5 | 10 | 5 | 25 | Moderate | 4.31 | 18 | Male | 100 | 1 | 49 | No |
| 2015 | Stutter | 1.62 | 2 | 2.00 | 3 | 4 | 1 | 10 | V. Light | 1.81 | 21 | Male | 100 | 3 | 39 | Yes |
| 2016 | Stutter | 2.40 | 4 | 1.80 | 2 | 4 | 3 | 13 | V. Light | 2.10 | 30 | Male | 80 | 1 | 36 | Yes |
| 2017 | Stutter | 2.42 | 4 | 3.32 | 4 | 6 | 1 | 15 | V. Light | 2.87 | 26 | Female | 100 | 1 | 48 | No |
| 2018 | Stutter | 18.40 | 8 | 6.21 | 6 | 6 | 2 | 22 | Light | 12.31 | 31 | Male | -60 | 3 | 46 | No |
| 2019 | Stutter | 29.46 | 9 | 23.62 | 9 | 14 | 9 | 41 | V. Severe | 26.54 | 25 | Male | 100 | 1 | 50 | Yes |
| 2020 | Stutter | 7.26 | 7 | 4.60 | 5 | 12 | 3 | 27 | Moderate | 5.93 | 19 | Male | 100 | 2 | 47 | Yes |
| 2021 | Stutter | 40.30 | 9 | 44.35 | 9 | 14 | 9 | 41 | V. Severe | 42.32 | 19 | Male | 100 | 1 | 45 | Yes |
| 2022 | Stutter | 9.82 | 7 | 16.15 | 8 | 12 | 5 | 32 | Severe | 12.99 | 26 | Female | -63.9 | 4 | 50 | Yes |
| 2023 | Stutter | 0.20 | 0 | 1.40 | 2 | 2 | 0 | 4 | None | 0.80 | 54 | Male | 100 | 5 | 45 | No |
| 2024 | Stutter | 5.60 | 6 | 4.60 | 5 | 8 | 1 | 20 | Light | 5.10 | 22 | Male | 100 | 1 | 59 | No |
| 2025 | Stutter | 22.92 | 9 | 12.40 | 8 | 14 | 6 | 37 | V. Severe | 17.66 | 29 | Male | 69.2 | 1 | 38 | No |
| 2026 | Stutter | 0.90 | 0 | 0.40 | 0 | 2 | 3 | 5 | None | 0.65 | 27 | Male | 100 | 5 | 40 | No |
| 2027 | Stutter | 16.70 | 8 | 16.20 | 8 | 12 | 8 | 36 | Severe | 16.45 | 30 | Male | 100 | 5 | 38 | Yes |

**Individual Stuttering Demographics** **–** Group was used for the main analyses. Percentage of stuttering was used for the supplementary analyses.
