# Supplementary material for: Adults who stutter lack the specialised pre-speech facilitation found in non-stutterers
Source: PLoS One. 2018 Oct 10;13(10):e0202634. doi: 10.1371/journal.pone.0202634 (PMC6179203; doi:10.1371/journal.pone.0202634)
Supplement: S3 Table — (DOCX) [file pone.0202634.s003.docx]

**S3 Table**

| Model | R | R² | Adjusted R² | Std. Error of the Estimate | Change Statistics | | | | |
| --- | --- | --- | --- | --- | --- | --- | --- | --- | --- |
|  |  |  |  |  | R² Change | F Change | df1 | df2 | Sig. F Change |
| 1 | 0.187 | 0.035 | 0.035 | 1.110 | 0.035 | 124.506 | 1 | 3441 | <0.001 |
| 2 | 0.199 | 0.040 | 0.039 | 1.108 | 0.005 | 5.715 | 3 | 3438 | 0.001 |

Model Summary for Experiment 2 – Additional statistical information pertaining to Table 3 in the text.
