# Supplementary material for: Adults who stutter lack the specialised pre-speech facilitation found in non-stutterers
Source: PLoS One. 2018 Oct 10;13(10):e0202634. doi: 10.1371/journal.pone.0202634 (PMC6179203; doi:10.1371/journal.pone.0202634)
Supplement: S4 Table — (DOCX) [file pone.0202634.s004.docx]

**S4 Table**

| Model | R | R² | Adjusted R² | Std. Error of the Estimate | Change Statistics | | | | |
| --- | --- | --- | --- | --- | --- | --- | --- | --- | --- |
|  |  |  |  |  | R² Change | F Change | df1 | df2 | Sig. F Change |
| 1 | 0.052 | 0.003 | 0.002 | 1.371 | 0.003 | 7.134 | 1 | 2676 | 0.008 |
| 2 | 0.097 | 0.009 | 0.008 | 1.367 | 0.007 | 6.143 | 3 | 2673 | <0.001 |

Model Summary for Experiment 3 – Additional statistical information pertaining to Table 4 in the text.
