# Supplementary material for: Adults who stutter lack the specialised pre-speech facilitation found in non-stutterers
Source: PLoS One. 2018 Oct 10;13(10):e0202634. doi: 10.1371/journal.pone.0202634 (PMC6179203; doi:10.1371/journal.pone.0202634)
Supplement: S5 Table — (DOCX) [file pone.0202634.s005.docx]

**S5 Table**

| Model | R | R² | Adjusted R² | Std. Error of the Estimate | Change Statistics | | | | |
| --- | --- | --- | --- | --- | --- | --- | --- | --- | --- |
|  |  |  |  |  | R² Change | F Change | df1 | df2 | Sig. F Change |
| 1 | 0.241 | 0.058 | 0.058 | 1.258 | 0.058 | 240.381 | 1 | 3914 | <0.001 |
| 2 | 0.249 | 0.062 | 0.061 | 1.256 | 0.004 | 5.966 | 3 | 3911 | <0.001 |

Model Summary for Experiment 1 – Additional statistical information pertaining to Table 7 in the text.
