# Supplementary material for: Adults who stutter lack the specialised pre-speech facilitation found in non-stutterers
Source: PLoS One. 2018 Oct 10;13(10):e0202634. doi: 10.1371/journal.pone.0202634 (PMC6179203; doi:10.1371/journal.pone.0202634)
Supplement: S1 Text — The constant (M = 0.711, SED = 0.117, p < .001), pre-speech interval (M = 0.980, SED = 0.306, p < .01) and group (M = -0.305, SED = 0.113, p < .01) were all lower than in the immediate condition, even after Bonferroni correction (sig. p < 0.0125). The interaction did not differ significantly (M = -0.642, SED = 0.427, p = .133). (DOCX) [file pone.0202634.s008.docx]

**S1 Text Appendix (to Table 5) – Specific statistical analysis of the comparison between experiments 1 and 2**

The constant (*M* = 0.711, *SED* = 0.117, *p* < .001), pre-speech interval (*M* = 0.980, *SED* = 0.306, *p* < .01) and group (*M* = -0.305, *SED* = 0.113, *p* < .01) were all lower than in the *immediate* condition, even after Bonferroni correction (sig. p < 0.0125). The interaction did not differ significantly (*M* = -0.642, *SED* = 0.427, *p* = .133).
