# Supplementary material for: Adults who stutter lack the specialised pre-speech facilitation found in non-stutterers
Source: PLoS One. 2018 Oct 10;13(10):e0202634. doi: 10.1371/journal.pone.0202634 (PMC6179203; doi:10.1371/journal.pone.0202634)
Supplement: S2 Text — The constant (M = 0.617, SED = 0.133, p < .001) and pre-speech interval (M = 2.295, SED = 0.356, p < .001) were significantly smaller than in the first experiment, while group (M = -0.302, SED = 0.126, p = .0167) and the group-interval interaction (M = -1.059, SED = 0.503, p = .035) were marginally non-significant after Bonferroni correction (sig. p < 0.0125) (DOCX) [file pone.0202634.s009.docx]

**S2 Text Appendix (to Table 6) – Specific statistical analysis of the comparison between experiments 1 and 3**

The constant (*M* = 0.617, *SED* = 0.133, *p* < .001) and pre-speech interval (*M* = 2.295, *SED* = 0.356, *p* < .001) were significantly smaller than in the first experiment, while group (*M* = -0.302, *SED* = 0.126, *p* = .0167) and the group-interval interaction (*M* = -1.059, *SED* = 0.503, *p* = .035) were marginally non-significant after Bonferroni correction (sig. p < 0.0125)
