# Supplementary material for: Adults who stutter lack the specialised pre-speech facilitation found in non-stutterers
Source: PLoS One. 2018 Oct 10;13(10):e0202634. doi: 10.1371/journal.pone.0202634 (PMC6179203; doi:10.1371/journal.pone.0202634)
Supplement: S3 Text — As a side note, one significant value was unexpected–the correlation between State and Trial in the first experiment. This implies that there were more late stimulation states in the later trials, on average. However, due to the random order of presentation and the significant correlations between Reaction Time and both Pulse Condition and Trial, we felt that this did not impact the study adversely. (DOCX) [file pone.0202634.s010.docx]

**S3 Text Appendix (to Tables 10a-d) – Significance of Reaction Time with Pulse Condition and Trial**

As a side note, one significant value was unexpected – the correlation between State and Trial in the first experiment. This implies that there were more late stimulation states in the later trials, on average. However, due to the random order of presentation and the significant correlations between Reaction Time and both Pulse Condition and Trial, we felt that this did not impact the study adversely.
